# Connexin 43 Deficiency Is Associated with Reduced Myocardial Scar Size and Attenuated TGFβ1 Signaling after Transient Coronary Occlusion in Conditional Knock-Out Mice

**DOI:** 10.3390/biom10040651

**Published:** 2020-04-23

**Authors:** Laura Valls-Lacalle, Marta Consegal, Marisol Ruiz-Meana, Begoña Benito, Javier Inserte, Ignasi Barba, Ignacio Ferreira-González, Antonio Rodríguez-Sinovas

**Affiliations:** 1 Cardiovascular Diseases Research Group, Department of Cardiology, Vall d’Hebron University Hospital and Research Institute, Departament de Medicina, Universitat Autònoma de Barcelona, Pg. Vall d’Hebron 119-129, 08035 Barcelona, Spain; lvalls92@gmail.com (L.V.-L.); marta.consegal@vhir.org (M.C.); mruizmeana@gmail.com (M.R.-M.); begona.benito@vhir.org (B.B.); javier.inserte@vhir.org (J.I.); ignasibarba@gmail.com (I.B.); 2 Centro de Investigación Biomédica en Red sobre Enfermedades Cardiovasculares (CIBERCV), 28029 Madrid, Spain; 3 Centro de Investigación Biomédica en Red sobre Epidemiología y Salud Pública (CIBERESP), 28029 Madrid, Spain

**Keywords:** connexin 43, left ventricular remodeling, collagen, ischemia–reperfusion, myocardial infarct

## Abstract

Previous studies demonstrated a reduction in myocardial scar size in heterozygous Cx43^+/-^ mice subjected to permanent coronary occlusion. However, patients presenting with ST segment elevation myocardial infarction often undergo rapid coronary revascularization leading to prompt restoration of coronary flow. Therefore, we aimed to assess changes in scar size and left ventricular remodeling following transient myocardial ischemia (45 min) followed by 14 days of reperfusion using Cx43^fl/fl^ (controls) and Cx43^Cre-ER(T)/fl^ inducible knock-out (Cx43 content: 50%) mice treated with vehicle or 4-hydroxytamoxifen (4-OHT) to induce a Cre-ER(T)-mediated global deletion of the Cx43 floxed allele. The scar area (picrosirius red), measured 14 days after transient coronary occlusion, was similarly reduced in both vehicle and 4-OHT-treated Cx43^Cre-ER(T)/fl^ mice, compared to Cx43^fl/fl^ animals, having normal Cx43 levels (15.78% ± 3.42% and 16.54% ± 2.31% vs. 25.40% ± 3.14% and 22.43% ± 3.88% in vehicle and 4-OHT-treated mice, respectively, *p* = 0.027). Left ventricular dilatation was significantly attenuated in both Cx43-deficient groups (*p* = 0.037 for left ventricular end-diastolic diameter). These protective effects were correlated with an attenuated enhancement in pro-transforming growth factor beta 1 (TGFβ1) expression after reperfusion. In conclusion, our data demonstrate that Cx43 deficiency induces a protective effect on scar formation after transient coronary occlusion in mice, an effect associated with reduced left ventricular remodeling and attenuated enhancement in pro-TGFβ1 expression.

## 1. Introduction

Connexins are a family of proteins that constitute integral components of plasma membranes. Connexin 43 (Cx43) is the most ubiquitous connexin isoform, and is widely distributed in most tissues, including cardiac cells [1]. In the heart, connexins form aggregations of intercellular channels known as gap junctions, which are mainly located at cardiomyocyte poles [2]. Gap junctions constitute low resistance pathways that are essential to allow electrical current flow between connected cells [3]. In addition to electrical coupling, gap junctions also allow chemical communication between neighboring cells, and are involved in spreading of cell death during acute myocardial ischemia–reperfusion injury [4].

In this sense, previous studies have demonstrated that gap junctions may facilitate propagation of hypercontracture and cardiomyocyte death during reperfusion, by a mechanism involving Na^+^ passage between adjacent cells and subsequent Ca^2+^ influx secondary to Na^+^/Ca^2+^ exchange [5]. This gap junction-mediated spreading of damage contributes to the final extension of myocardial necrosis [6]. In agreement with this, the administration of the gap junction uncoupler heptanol at the onset of reperfusion has been demonstrated to reduce infarct size in several animal models [5]. Moreover, the protective effect of heptanol was extended to other gap junction uncouplers [7], and to other situations as hypoxia/reoxygenation [8]. These findings were further strengthened by data obtained in our group demonstrating that isolated hearts from transgenic mice lacking Cx43, either by replacement with Cx32, or by inducible knock-out, had smaller acute infarctions after one hour of reperfusion following transient global ischemia [9,10].

Acute myocardial infarction leads to adverse left ventricular remodeling and heart failure, a disabling disease that seriously affects the quality of life of those patients, worsens its prognosis and whose triggering mechanisms are not completely understood [11]. In this regard, the effects of Cx43 on postinfarction scar formation and left ventricular remodeling beyond the acute phase following myocardial ischemia are less known. It has been reported that reduced Cx43 expression in heterozygous Cx43^+/−^ mice attenuates myocardial infarct size or collagen deposition, both in the infarcted and non-infarcted cardiac regions, several weeks after permanent coronary occlusion [12,13]. However, these studies are difficult to interpret as they used permanent coronary ligations, a maneuver expected to cause transmural infarctions [14,15] and that is far from the clinics, where most patients presenting with ST-segment elevation myocardial infarction (STEMI) are quickly subjected to coronary revascularization, most often by primary percutaneous coronary intervention [16,17]. Furthermore, as homozygous Cx43^−/−^ knock-out animals are not viable and die soon after birth, most studies analyzing the role of Cx43 on left ventricular remodeling (and also on acute ischemia–reperfusion injury) have used heterozygous Cx43^+/−^ mice models [12,13]. These animals show a partial Cx43 deficiency that is present from birth, and that is possibly associated with compensatory changes. In this sense, use of an inducible knock-out model for Cx43 would be advantageous, as it is possible to obtain an almost complete Cx43 deficiency in adult animals.

In this context, the aims of this work were to analyze changes in scar size and in left ventricular remodeling following transient myocardial ischemia followed by reperfusion in a Cx43^Cre-ER(T)/fl^ mice model with partial (50%) or almost complete Cx43 deficiency after induction with 4-hydroxytamoxifen (4-OHT). Furthermore, we investigated how these changes were correlated with altered expression of selected markers related to cardiac remodeling and fibrosis, including transforming growth factor beta 1 (TGFβ1) and nuclear factor Kappa-light-chain-enhancer of activated B cells (NF-κB).

## 2. Materials and Methods

The present study conforms to the NIH Guide for the Care and Use of Laboratory Animals (NIH publications N°. 85-23, revised 1996), and was performed in accordance with European legislation (Directive 2010/63/UE). The study was approved by the Ethics Committee of Vall d’Hebron Hospital and Research Institute (CEEA 35.17).

### 2.1. Animals

Cx43 deficiency was achieved by 4-OHT treatment in adult Cx43^Cre-ER(T)/fl^ mice, as previously described [10,18]. Cx43^Cre-ER(T)/fl^ animals were developed by Eckardt et al. [18], and have a C57BL/6J background. In Cx43^Cre-ER(T)/fl^ animals, the coding region of one of the Cx43 alleles was replaced by Cre-ER(T), a fusion construct of the Cre recombinase and a mutated domain of the human estrogen receptor. Cre recombinase is activated after administration of 4-OHT, whereas ER(T) is insensitive to the natural ligand β-estradiol. Cre activation induces a global deletion of Cx43, after recognition of loxP sites flanking the second Cx43 allele, whereas no compensation by overexpression of other connexin isoforms has been demonstrated [10,18,19,20]. Cx43^Cre-ER(T)/fl^ mice and their corresponding controls (Cx43^fl/fl^) were injected intraperitoneally with vehicle (castor oil) or 3 mg/day 4-OHT suspended in plant oil for 5 consecutive days.

### 2.2. Experimental Protocol

Thirty-five adult male mice (10–12 weeks, 30–35 g) were artificially ventilated (SAR-830/P Ventilator, CWE Inc., Ardmore, PA, USA) after anesthesia with ketamine (50 mg/Kg, IP) and sodium pentobarbital (40 mg/Kg, IP) [21]. Then, animals were placed on an adjustable heating pad to maintain core temperature between 36 and 37 °C, and the heart was exposed through a lateral thoracotomy at the fourth intercostal space. The left anterior descending coronary artery (LAD) was occluded with a 6/0 silk snare (Ethicon Endo-surgery, Cincinnati, OH, USA), placed 1 mm distal to the left atrial appendage. All animals were subjected to 45 min of LAD occlusion followed by 14 days of reperfusion. ST segment elevation at the electrocardiogram and visual pallor of distal myocardium were used as markers of successful LAD occlusion. After reperfusion, the occluding snare was kept in place to allow later determination of ligature position. Following surgery all animals were treated with buprenorphine (0.05 mg/Kg every 6 h, subcutaneously) during the first 48 h. Treatment with vehicle or 4-OHT began 24 h after infarction. Cx43 deletion in Cx43^Cre-ER(T)/fl^ animals treated with 4-OHT is progressive and is not apparent until about 5–7 days after first injection [10,20]. At the end of the experiment, animals were sacrificed by a sodium pentobarbital overdose (150 mg/Kg, IP).

### 2.3. Myocardial Scar Size

The histological extent of the myocardial scar 14 days after reperfusion was calculated as the percentage of the fibrotic area to left ventricular area, as previously described [22]. Hearts were quickly excised and, after removal of both atria and great vessels, the apical region, just below the snare, was fixed overnight with 4% paraformaldehyde, embedded in paraffin and cut in 4 µm sections. Histological sections were stained with picrosirius red (Sigma-Aldrich, St. Louis, MO, USA), scanned and evaluated using ImageProPlus software (Media Cybernetics, Rockville, MD, USA).

### 2.4. Transthoracic Echocardiography

Echocardiographic measurements were performed in Cx43^fl/fl^ and Cx43^Cre-ER(T)/fl^ mice, both at baseline and after 14 days of reperfusion, with a Vivid q portable ultrasound system, using a ILS 12 MHz transducer (GE Healthcare, WI, USA) after light anesthesia with isoflurane (1%–1.5%). Ejection fraction (EF), left ventricular end-diastolic internal diameter (LVEDD), left ventricular end-systolic internal diameter (LVESD), interventricular septum thickness (IVS) and posterior wall thickness (LVPW) were measured in M-mode recordings. Fractional shortening (FS) was calculated as (LVEDD-LVESD)/LVEDD × 100.

### 2.5. Western Blot

Snap-frozen myocardium was homogenized (Diax 600 homogenizer, Heidolph, Schwabach, Germany) in homogenization RIPA buffer (in mmol/L: NaCl 150, Trizma base 50, Triton X-100 1%, sodium deoxycholate 0.5%, SDS 0.1%, Tween 20 0.1%, sodium fluoride 5, sodium orthovanadate 1 and a protease cocktail inhibitor (1%), pH 8.0). Protein extracts from mice hearts were obtained from the supernatant after centrifugation at 1600 g for 10 min (4 °C), and were electrophoretically separated on 10% polyacrylamide gels. Expression of NF-κB p65, Smad2/3, phosphorylated Smad2/3 (Thr8), TGFβ1 and connective tissue growth factor (CTGF) was analyzed by Western blot according to standard procedures [23]. Glyceraldehyde-3-phosphate dehydrogenase (GAPDH) was used as loading control. The degree of activation of Smad2/3 was defined as the ratio between phosphorylated and total forms. Immunoreactive bands were detected using the SuperSignal West Dura Extended Duration Substrate (Pierce, Rockford, IL, USA) and densitometrically analyzed.

The following antibodies were used: rabbit anti-NF-κB p65 (#ab16502, Abcam, dilution 1:1000), anti-Smad2/3 (total forms: #sc398844 (raised in mice), Santa Cruz Biotechnology, Dallas, TX, USA, 1:500; phosphorylated forms (Thr8): #TA325852 (raised in rabbit), Origene, Rockville, MD, USA, 1:1000), rabbit anti-TGFβ1 (#ab92486, Abcam, Cambridge, UK, dilution 1:1000), rabbit anti-CTGF (#ab6992, abcam, dilution 1:1000) and mouse anti-GAPDH (#GTX627408, GeneTex, Irvine, CA, USA, 1:5000).

### 2.6. Statistics

Data are expressed as mean ± standard error of the mean (SEM). Differences were assessed with the Student’s *t* test, analysis of variance (ANOVA) and Tukey post-test, two-way ANOVA or repeated measures ANOVA (MANOVA) as needed. Differences were considered significant when *p* < 0.05.

## 3. Results

### 3.1. Effects of Cx43 Deficiency on Myocardial Scar Size after Transient Coronary Occlusion

**Figure 1:**
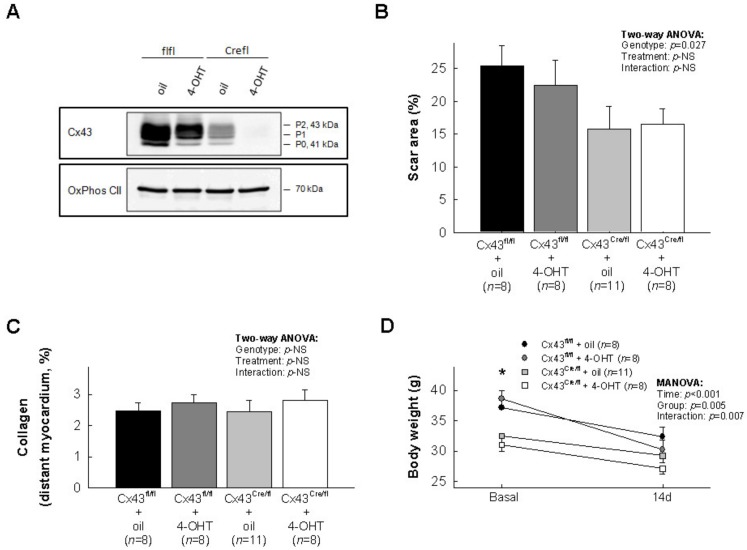
Cx43 expression under baseline conditions, assessed by conventional Western blot, in hearts from the four experimental groups. Mitochondrial complex II (OxPhos CII) was used as loading control (**A**). Effects of transient coronary occlusion on scar area (**B**), collagen volume fraction in distant myocardium (**C**) and body weight (**D**) in Cx43^fl/fl^ and Cx43^Cre-ER(T)/fl^ mice treated with oil or 4-OHT, and submitted to 45 min of left anterior descending coronary artery (LAD) occlusion followed by 14 days of reperfusion. Statistical analysis was performed by two-way ANOVA or repeated measures ANOVA, as depicted. * (*p* < 0.05) indicates significant differences between both genotypes (ANOVA and Tukey tests).

Cx43 expression in the four experimental groups is shown in Figure 1A. The scar area, determined by picrosirius red staining 14 days after transient coronary occlusion, was similarly reduced in both oil (50% Cx43 expression) and 4-OHT (<5% Cx43 expression)-treated Cx43^Cre-ER(T)/fl^ mice, as compared with Cx43^fl/fl^ animals, with normal Cx43 levels (16.10% ± 2.16% for both Cx43-deficient animals grouped vs. 23.91% ± 2.44% for both Cx43^fl/fl^ mice grouped, *p* = 0.022, Student’s *t* test; Figure 1B). Two-way ANOVA analysis demonstrated a significant effect of genotype, whereas no significant effects of treatment (oil vs. 4-OHT) or interactions between treatment and genotype were detected (Figure 1B). Collagen volume fraction in distant myocardium was low in all groups 14 days after myocardial infarction, with no differences between groups (Figure 1C).

Reduction of body weight has been previously associated with worse clinical outcomes after myocardial infarction [24]. As shown in Figure 1D, myocardial infarction was associated with a reduction of body weight in all groups, but this effect was significantly higher in Cx43^fl/fl^ animals (*p* = 0.007 for interaction between time and group; Figure 1D).

### 3.2. Effects of Cx43 Deficiency on Post-Infartion Left Ventricular Remodeling

**Figure 2:**
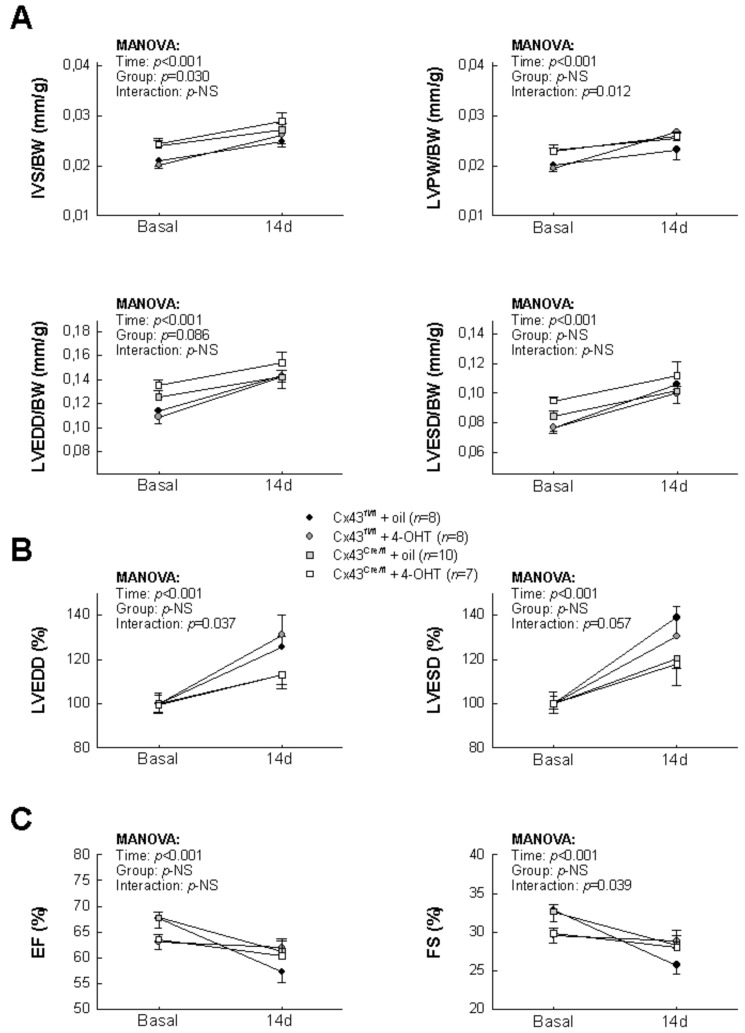
Echocardiographic assessment of post-infartion left ventricular remodeling in Cx43-deficient mice. (**A**) Changes in interventricular septum thickness (IVS), left ventricular posterior wall thickness (LVPW), left ventricular end-diastolic internal diameter (LVEDD) and left ventricular end-systolic internal diameter (LVESD) in Cx43^Cre-ER(T)/fl^ and Cx43^fl/fl^ mice, treated with oil or 4-OHT, and submitted to 45 min of LAD occlusion and 14 days of reperfusion. Data have been normalized by body weight (BW). (**B**) Changes in LVEDD and LVESD, expressed as percentage of baseline values in the same groups of animals. Repeated measures ANOVA demonstrated a significant interaction between time and group in both cases. (**C**) Changes in ejection fraction (EF) and fractional shortening (FS) in all experimental groups. Symbols are common for all figure panels and its interpretation is shown between panels A and B.

Table 1 shows absolute values for IVS, LVPW LVEDD and LVESD, both at baseline and 14 days after reperfusion, in Cx43^Cre-ER(T)/fl^ and Cx43^fl/fl^ mice subjected to transient coronary occlusion. As shown in Figure 2A, animals subjected to myocardial ischemia–reperfusion depicted enhancements in IVS, LVPW LVEDD and LVESD, expressed in relation to body weight, 14 days after coronary occlusion, that were not significantly different between experimental groups. However, when changes in cardiac dimensions were expressed as percentage of baseline values, Cx43-deficient animals had an attenuated increase in LVEDD respect to Cx43^fl/fl^ mice (*p* = 0.015 for interaction between time and group, Figure 2B), supporting a lesser left ventricular remodeling. A similar trend was observed for LVESD although differences did not reach statistical significance (*p* = 0.057 for interaction). On the other hand, EF and FS were reduced in all groups, with no clear differences between them (Figure 2C).

### 3.3. Changes in Expression of Selected Fibrotic and Remodeling Markers 14 Days after Transient Coronary Occlusion

**Figure 3:**
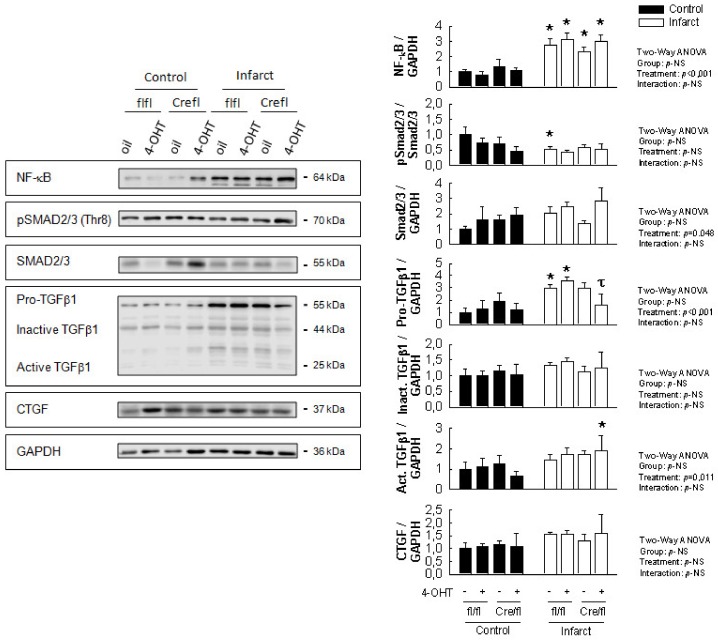
Changes in the expression of NF-κB, Smad2/3, pSmad2/3, TGFβ1 and CTGF in hearts from Cx43^Cre-ER(T)/fl^ and Cx43^fl/fl^ mice, treated with oil or 4-OHT that have been subjected to 45 min of LAD occlusion and 14 days of reperfusion. Right panels show the corresponding protein quantification of 4 different Western blots. Left panels show original representative Western blots. Two-way ANOVA shows differences for group, treatment (control vs. infarction) and interaction between both factors. * (*p* < 0.05) indicates significant differences vs. the corresponding control group. τ (*p* < 0.05) indicates significant differences between infarcted 4-OHT-treated Cx43^Cre-ER(T)/fl^ mice and both infarcted Cx43^fl/fl^ groups (ANOVA and Tukey tests).

Two-way ANOVA analysis demonstrated a significantly enhanced myocardial expression of NF-κB, pro-TGFβ1 and the active form of TGFβ1, together with a marginal increase in total Smad2/3, in animals subjected to transient coronary occlusion followed by reperfusion for 14 days (Figure 3). However, when compared with their corresponding non-infarcted control groups, the increase in pro-TGFβ1 seen in Cx43-deficient animals (especially in those treated with 4-OHT, showing a marked reduction in Cx43 expression) was lower, and did not reach statistical significance (Figure 3). Furthermore, a significant difference for pro-TGFβ1 was observed between infarcted 4-OHT-treated Cx43^Cre-ER(T)/fl^ mice and both infarcted Cx43^fl/fl^ groups. No changes were observed in any group in the expression of CGTF, inactive TGFβ1 or pSmad2/3.

## 4. Discussion

The present study demonstrates that cardiac Cx43 deficiency in conditional Cx43^Cre-ER(T)/fl^ mice induces a protective effect against scar formation after transient coronary occlusion, as demonstrated by a reduction in the histological extent of the myocardial scar 14 days after reperfusion. This protective effect was associated with attenuated LV dilatation, as assessed by echocardiography, and reduced body weight loss, both suggestive of improved left ventricular remodeling. Moreover, these protective effects were associated with an attenuated enhancement in the expression of pro-TGFβ1 after reperfusion, which could explain, at least in part, these findings.

Previous studies have shown that a reduction in Cx43 expression in heterozygous Cx43^+/-^ mice attenuates myocardial infarct size, as assessed by histological Masson’s trichrome staining of ventricular slices, both during the healing phase (8 days after surgery) and in fully healed infarcts (10 weeks after occlusion), as compared with Cx43^+/+^ wild-type animals [13]. Similarly, an attenuated collagen accumulation was observed in the same animal model, both in the infarcted and non-infarcted cardiac regions, 6 days and 4 weeks after coronary occlusion [12]. However, in these two studies, animals were subjected to permanent coronary ligatures, a maneuver that should cause a transmural infarction [14,15] but is far from the clinical setting, where patients presenting with STEMI rapidly undergo coronary revascularization [16,17]. We believe that our model of transient ischemia followed by reperfusion recapitulates more reliably the clinical context present in patients with STEMI, and therefore, has greater translational value.

Here, we demonstrated that both a mild and a marked Cx43 deficiency after myocardial infarction was associated with reduced scar, and that this protective effect was of similar magnitude in both cases. Cx43^Cre-ER(T)/fl^ mice, when treated with vehicle (oil), express about 50% of normal Cx43 content, whereas administration of 4-OHT induces a gradual reduction of Cx43 levels to become almost undetectable 14 days after first induction [10,18,25]. Although one could expect that Cx43^Cre-ER(T)/fl^ mice treated with 4-OHT would show even a higher reduction in scar size, this was not the case. The reason for this might be due to the fact that reduction in Cx43 levels in this model is progressive and, short after infarct induction, Cx43 levels are still around 50%, taking about one week to become significantly decreased [10,18,25]. We are, thus, restricted by the temporal limitations imposed by our animal model, which shows a high mortality 14 days after 4-OHT induction, mostly due to malignant arrhythmias. Cx43 is a key element in cell-to-cell communication and its loss may favor reentrant circuits facilitating arrhythmogenicity [10,18,25]. It is worth to mention, however, that in three additional animals we attempted to modify the timings of our experiments, administrating 4-OHT one week before transient coronary ligation and assessing scar size 7 days after. However, these animals died within 24 h of coronary occlusion, again probably due to malignant ventricular arrhythmias in relation to the reduced Cx43 levels.

The protective effect of Cx43 deficiency on collagen deposition after transient coronary occlusion is similar to that described in other tissues. Thus, previous studies conducted in different animal species have shown that downregulation of Cx43 levels improves the rate of wound healing in the skin, causing smaller scars [26,27,28,29,30]. Similarly, the Cx43 mimetic peptide Gap27 has been demonstrated to promote healing of superficial epithelial corneal wounds [31]. Our findings are also in agreement with the results obtained in hearts submitted to cryoinjury that demonstrated an attenuation of left ventricular remodeling and scar area after the administration of the carboxiterminal mimetic peptide αCT1, which competitively inhibits the interaction of endogenous Cx43 with zonula occludens-1 [27,32]. In contrast, others have shown that a mild Cx43 deficiency in Cx43^Cre-ER(T)/fl^ mice, expressing 50% of normal Cx43 content, is associated with an increase in collagen deposition after pressure overload induced by transverse aortic constriction [33] or angiotensin II treatment [25]. However, we have previously suggested that this increase in collagen deposition observed after angiotensin II treatment in mild Cx43-deficient mice is probably independent of Cx43, as it is not seen in Cx43^+/-^ mice, having a similar Cx43 depletion, and is reverted when Cx43^Cre-ER(T)/fl^ animals were treated with 4-OHT [25]. Thus, these results, obtained in a different model of left ventricular remodeling (angiotensin II treatment vs. transient coronary occlusion in our present study) also support the notion that Cx43 deficiency exerts a protective action against collagen deposition.

The reduction in scar size seen in our Cx43-deficient mice is associated with an attenuated enhancement in the expression of pro-TGFβ1 after reperfusion. TGFβ1 plays a key role in cardiac fibrosis and remodeling [34]. It is, thus, tempting to speculate that a reduction in TGFβ1 signaling in our Cx43-deficient model might explain, at least in part, our findings. In fact, previous studies have also shown an association between Cx43 deficiency and a reduction in TGFβ signaling [12]. However, and contrary to that study, changes in pro-TGFβ1 do not translate, in our present work, into changes in Smad2/3 levels or in its degree of phosphorylation. Previous studies have demonstrated that the profibrotic cardiovascular effects of TGFβ1 are largely due to activation of the Smad signaling pathway [35]. We do not have a clear explanation for this discrepancy. However, one possibility lies in the fact that upon binding of TGFβ1 to its specific membrane receptors, it may induce both the canonical Smad-dependent signaling pathway, as well as pathways without direct involvement of Smad proteins, which activate signaling molecules like mitogen-activated protein kinase or small GTPases [36]. This possibility deserves, thus, future investigations.

We have previously demonstrated that cardiac fibroblasts isolated from Cx43^Cre-ER(T)/fl^ mice treated with 4-OHT had, under baseline conditions, a marked reduction in expression of α-smooth muscle actin and SM22α, two markers of cell differentiation [25]. Simultaneously, these fibroblasts depicted an abnormal phenotype, including reduced cell size and highly refringent nuclei. Furthermore, hearts from these animals had an enhanced vimentin expression, suggestive of higher fibroblast content [25]. All these changes occurring at the fibroblast/myofibroblast level may explain, at least in part, the reduced scar area seen in this group after transient coronary occlusion. In addition, hearts from these animals had, already under baseline conditions, an enhanced inflammatory response, with increased expression of IL-6 and NOX2 mRNA and enhanced LAMP2/Mac3 (a macrophage marker) and MMP9 levels, as assessed by immunohistochemistry [25]. This enhanced inflammatory response, and especially the increased MMP9 levels may help to explain a reduced scar area in this group.

## 5. Conclusions

In conclusion, our present study demonstrates a protective action of Cx43 deficiency on scar formation after transient coronary occlusion in mice, an effect that is associated with attenuated left ventricular remodeling and reduced enhancement in pro-TGFβ1 expression.

## Figures and Tables

**Table 1:** Absolute values (in mm) for interventricular septum thickness (IVS), left ventricular posterior wall thickness (LVPW), left ventricular end-diastolic internal diameter (LVEDD) and left ventricular end-systolic internal diameter (LVESD), both at baseline and 14 days after reperfusion, in Cx43^Cre-ER(T)/fl^ and Cx43^fl/fl^ mice subjected to transient coronary occlusion.

	IVS		LVPW		LVEDD		LVESD	
	Baseline	14d	Baseline	14d	Baseline	14d	Baseline	14d
Cx43^fl/fl^ + oil	0.77 ± 0.02	0.80 ± 0.03	0.74 ± 0.02	0.73 ± 0.04	4.21 ± 0.16	4.59 ± 0.20 *	2.82 ± 0.09	3.42 ± 0.19 *
(*n* = 8)								
Cx43^fl/fl^ + 4-OHT	0.77 ± 0.01	0.77 ± 0.02	0.75 ± 0.03	0.79 ± 0.01	4.17 ± 0.11	4.23 ± 0.17	2.93 ± 0.08	2.97 ± 0.15
(*n* = 8)								
Cx43^Cre/fl^ + oil	0.77 ± 0.02	0.78 ± 0.02	0.74 ± 0.02	0.73 ± 0.02	4.02 ± 0.11	4.05 ± 0.10	2.70 ± 0.08	2.90 ± 0.09 *
(*n* = 10)								
Cx43^Cre/fl^ + 4-OHT	0.76 ± 0.02	0.79 ± 0.04	0.72 ± 0.02	0.71 ± 0.02	4.26 ± 0.26	4.26 ± 0.26	2.98 ± 0.15	3.09 ± 0.25
(*n* = 7)								

* (*p* < 0.05) indicates significant differences vs. corresponding baseline value (paired Student’s *t* test). Abbreviations: 14d: 14 days; 4-OHT: 4-hydroxytamoxifen; IVS: interventricular septum thickness; LVEDD: left ventricular end-diastolic internal diameter; LVESD: left ventricular end-systolic internal diameter; LVPW: left ventricular posterior wall thickness.

## References

[1] Lambiase P.D., Tinker A. (2014). Connexins in the heart. Cell Tissue Res..

[2] Sosinsky G.E., Nicholson B. (2005). Structural organization of gap junction channels. Biochimica et Biophysica Acta (BBA) Biomembranes.

[3] Kléber A.G., Saffitz J.E. (2014). Role of the intercalated disc in cardiac propagation and arrhythmogenesis. Front. Physiol..

[4] García-Dorado D., Rodriguez-Sinovas A., Ruiz-Meana M. (2004). Gap junction-mediated spread of cell injury and death during myocardial ischemia–reperfusion. Cardiovasc. Res..

[5] Garcia-Dorado D., Inserte J., Ruiz-Meana M., Gonzaález M.A., Solares J., Juliá M., Barrabés J.A., Soler-Soler J. (1997). Gap Junction Uncoupler Heptanol Prevents Cell-to-Cell Progression of Hypercontracture and Limits Necrosis During Myocardial Reperfusion. Circulation.

[6] Ruiz-Meana M., Garcia-Dorado D., Hofstaetter B., Piper H.M., Soler-Soler J. (1999). Propagation of cardiomyocyte hypercontracture by passage of Na(+) through gap junctions. Circ. Res..

[7] Rodriguez-Sinovas A., García-Dorado D., Ruiz-Meana M., Soler-Soler J. (2004). Enhanced effect of gap junction uncouplers on macroscopic electrical properties of reperfused myocardium. J. Physiol..

[8] Rodriguez-Sinovas A., García-Dorado D., Ruiz-Meana M., Soler-Soler J. (2006). Protective effect of gap junction uncouplers given during hypoxia against reoxygenation injury in isolated rat hearts. Am. J. Physiol. Circ. Physiol..

[9] Rodriguez-Sinovas A., Sánchez J.A., González-Loyola A., Barba I., Morente M., Aguilar R., Agulló E., Miró-Casas E., Esquerda N., Ruiz-Meana M. (2010). Effects of substitution of Cx43 by Cx32 on myocardial energy metabolism, tolerance to ischaemia and preconditioning protection. J. Physiol..

[10] Sánchez J.A., Rodriguez-Sinovas A., Barba I., Miró-Casas E., Fernández-Sanz C., Ruiz-Meana M., Alburquerque-Béjar J.J., García-Dorado D. (2013). Activation of RISK and SAFE pathways is not involved in the effects of Cx43 deficiency on tolerance to ischemia–reperfusion injury and preconditioning protection. Basic Res. Cardiol..

[11] Bhatt A.S., Ambrosy A.P., Velazquez E.J. (2017). Adverse Remodeling and Reverse Remodeling After Myocardial Infarction. Curr. Cardiol. Rep..

[12] Zhang Y., Wang H., Kovacs A., Kanter E.M., Yamada K.A. (2009). Reduced expression of Cx43 attenuates ventricular remodeling after myocardial infarction via impaired TGF-beta signaling. Am. J. Physiol. Circ. Physiol..

[13] Kanno S., Kovacs A., Yamada K.A., Saffitz J.E. (2003). Connexin43 as a determinant of myocardial infarct size following coronary occlusion in mice. J. Am. Coll. Cardiol..

[14] Nofi C., Bogatyryov Y., Dedkov E. (2017). Preservation of Functional Microvascular Bed Is Vital for Long-Term Survival of Cardiac Myocytes Within Large Transmural Post-Myocardial Infarction Scar. J. Histochem. Cytochem..

[15] Frangogiannis N.G. (2015). Pathophysiology of Myocardial Infarction. Compr. Physiol..

[16] Zhao Y., Zhu Y., Hu C., Du Y., Liu Y., Liu J., Zhang J., Cheng G., Han H., Zhao Q. (2019). Significant association between serum resistin and hypersensitive troponin I levels in patients with a first ST-segment elevation myocardial infarction. Nat. Rev. Dis. Prim..

[17] Kristensen S.D., Laut K.G., Fajadet J., Kaifoszova Z., Kala P., Di Mario C., Wijns W., Clemmensen P., Agladze V., Antoniades L. (2014). Reperfusion therapy for ST elevation acute myocardial infarction 2010/2011: Current status in 37 ESC countries. Eur. Heart J..

[18] Eckardt D. (2004). Functional role of connexin43 gap junction channels in adult mouse heart assessed by inducible gene deletion. J. Mol. Cell. Cardiol..

[19] Van Rijen H.V., Eckardt M., Degen J., Theis M., Ott T., Willecke K., Jongsma H.J., Opthof T., De Bakker J.M. (2004). Slow Conduction and Enhanced Anisotropy Increase the Propensity for Ventricular Tachyarrhythmias in Adult Mice with Induced Deletion of Connexin43. Circulation.

[20] Sánchez J.A., Rodriguez-Sinovas A., Fernández-Sanz C., Ruiz-Meana M., Garcia-Dorado D. (2011). Effects of a reduction in the number of gap junction channels or in their conductance on ischemia-reperfusion arrhythmias in isolated mouse hearts. Am. J. Physiol. Circ. Physiol..

[21] Shekarforoush S., Fatahi Z., Safari F. (2015). The effects of pentobarbital, ketamine–pentobarbital and ketamine–xylazine anesthesia in a rat myocardial ischemic reperfusion injury model. Lab. Anim..

[22] Poncelas M., Inserte J., Aluja D., Hernando V., Vilardosa Ú., García-Dorado D. (2017). Delayed, oral pharmacological inhibition of calpains attenuates adverse post-infarction remodelling. Cardiovasc. Res..

[23] Boengler K., Dodoni G., Rodriguez-Sinovas A., Cabestrero A., Ruiz-Meana M., Gres P., Konietzka I., Lopez-Iglesias C., García-Dorado D., Di Lisa F. (2005). Connexin 43 in cardiomyocyte mitochondria and its increase by ischemic preconditioning. Cardiovasc. Res..

[24] Jimenez F.L., Wu C.O., Tian X., O’Connor C., Rich M.W., Burg M.M., Sheps D., Raczynski J., Somers V.K., Jaffe A.S. (2008). Weight Change after Myocardial Infarction—the Enhancing Recovery in Coronary Heart Disease patients (ENRICHD) Experience. Am. Hear. J..

[25] Valls-Lacalle L., Negre-Pujol C., Rodríguez C., Varona S., Valera-Cañellas A., Consegal M., Martínez-González J., Rodríguez-Sinovas A. (2019). Opposite Effects of Moderate and Extreme Cx43 Deficiency in Conditional Cx43-Deficient Mice on Angiotensin II-Induced Cardiac Fibrosis. Cells.

[26] Qiu C., Coutinho P., Frank S., Franke S., Law L.-Y., Martin P., Green C., Becker D. (2003). Targeting connexin43 expression accelerates the rate of wound repair. Curr. Boil..

[27] Ongstad E.L., O’Quinn M.P., Ghatnekar G.S., Yost M.J., Gourdie R.G. (2013). A Connexin43 Mimetic Peptide Promotes Regenerative Healing and Improves Mechanical Properties in Skin and Heart. Adv. Wound Care.

[28] Ghatnekar G.S., O’Quinn M.P., Jourdan L.J., Gurjarpadhye A., Draughn R.L., Gourdie R.G. (2009). Connexin43 carboxyl-terminal peptides reduce scar progenitor and promote regenerative healing following skin wounding. Regen. Med..

[29] Gilmartin D.J., Soon A., Thrasivoulou C., Phillips A.R.J., Jayasinghe S., Becker D.L. (2016). Sustained Release of Cx43 Antisense Oligodeoxynucleotides from Coated Collagen Scaffolds Promotes Wound Healing. Adv. Heal. Mater..

[30] Montgomery J., Ghatnekar G., Grek C.L., Moyer K., Gourdie R.G. (2018). Connexin 43-Based Therapeutics for Dermal Wound Healing. Int. J. Mol. Sci..

[31] Elbadawy H., Mirabelli P., Xeroudaki M., Parekh M., Bertolin M., Breda C., Cagini C., Ponzin D., Lagali N., Ferrari S. (2016). Effect of connexin 43 inhibition by the mimetic peptide Gap27 on corneal wound healing, inflammation and neovascularization. Br. J. Pharmacol..

[32] O’Quinn M.P., Palatinus J.A., Harris B.S., Hewett K.W., Gourdie R.G. (2011). A Peptide Mimetic of the Connexin43 Carboxyl-Terminus Reduces Gap Junction Remodeling and Induced Arrhythmia Following Ventricular Injury. Circ. Res..

[33] Jansen J.A., Van Veen T.A., De Jong S., Van Der Nagel R., Van Stuijvenberg L., Driessen H., Labzowski R., Oefner C.M., Bosch A.A., Nguyen T.Q. (2012). Reduced Cx43 Expression Triggers Increased Fibrosis Due to Enhanced Fibroblast Activity. Circ. Arrhythmia Electrophysiol..

[34] Leask A. (2015). Getting to the Heart of the Matter. Circ. Res..

[35] Ruiz-Ortega M., Rodriguez-Vita J., Sanchez-Lopez E., Carvajal G., Egido J. (2007). TGF-β signaling in vascular fibrosis. Cardiovasc. Res..

[36] Horbelt D., Denkis A., Knaus P. (2012). A portrait of Transforming Growth Factor beta superfamily signalling: Background matters. Int. J. Biochem. Cell Biol..

